# Implementation of a Quit Smoking Programme in Community Adult Mental Health Services–A Qualitative Study

**DOI:** 10.3389/fpsyt.2018.00670

**Published:** 2018-12-21

**Authors:** Annette Burns, Margaret Webb, Greg Stynes, Tom O'Brien, Daniela Rohde, Judith Strawbridge, Luke Clancy, Frank Doyle

**Affiliations:** ^1^Department of Psychology, Royal College of Surgeons in Ireland, Dublin, Ireland; ^2^EVE, Health Service Executive, Dublin, Ireland; ^3^School of Pharmacy, Royal College of Surgeons, Dublin, Ireland; ^4^TobaccoFree Research Institute, Dublin Institute of Technology, Dublin, Ireland

**Keywords:** smoking, smoking cessation, mental illness, mental health, community, qualitative analysis, implementation, intervention

## Abstract

Little is known about the experiences of people with severe mental health difficulties in smoking cessation interventions. This study aimed to review the implementation of a smoking cessation programme across 16 community mental health day services. The aim was to establish the experience from both service user and facilitator perspectives and refine implementation for future groups. In-depth interviews were conducted with 20 service users and four focus groups held with 17 facilitators. Thematic analysis was used to analyse the data for emergent themes in relation to key enablers and barriers to implementation. Data from service users and facilitators revealed that implementation was enabled by an open and engaged recruitment approach; the resourcefulness of facilitators; programme materials and group-based format; combining the cessation programme with other and broader health initiatives; and participants' motivations, including health and money. Barriers included the structure of the service; the lack of a joined-up approach across the health services; literacy issues and the serial/logical process assumed by the programme. Barriers perceived as more specific to those with mental health difficulties included the use of smoking as a coping mechanism, lack of alternative activities/structure and lack of consistent determination. The tobacco free policy, implemented shortly before the programme, interestingly emerged as both a barrier and an enabler. In conclusion, although this group-based cessation programme in community mental health settings was well-received overall, a number of key barriers persist. A joined-up approach which addresses the culture of smoking in mental health settings, inconsistencies in smoking policies, and provides consistent cessation support, is needed. Care needs to be taken with the timing as overall it may not be helpful to introduce a new smoking cessation programme at the same time as a tobacco free policy.

## Introduction

The increased prevalence of smoking among those with mental health difficulties (MHDs) has been well-established (1–4), as has its impact in terms of tobacco-related morbidity (4–11) and mortality (12–14). Evidence indicates that cessation may be more difficult for those with mental health difficulties (4, 15, 16), but mental health has been shown to improve in those with and without psychiatric disorders post quitting (17). Smoking cessation programmes in community mental health settings meanwhile remain understudied (18, 19). The studies which have been conducted tend to focus on quantitative methods (18, 20–24), brief outcomes data (18, 22–26), and had small samples (<30) (20, 22, 25).

Studies taking a more in-depth approach often include qualitative data for facilitators only (27, 28), omitting service user views (27), or rely on surveys with no in-depth exploration of their experience included (28). Conversely, Rae et al. provided rich qualitative data of the experience of those with severe mental illness on cessation interventions, yet included no data detailing the experiences of facilitators (29). The only prior study to qualitatively evaluate a cessation intervention for those with MHDs including both service user and facilitator perspectives was limited by including only service users who requested treatment, a sample of just 3 facilitators and no implementation data (30).

The available evidence does suggest that people with MHDs want to quit smoking and would benefit from doing so (17, 31, 32), that community programmes are able to reach a high proportion of smokers, and when tailored can be effective for those with mental illness for quitting (18, 24), or reducing smoking (21). Qualitative data from a provider perspective have revealed that a tailored tobacco cessation was feasible and well-received (28). Beyond implementation studies, qualitative data in relation to experiences of cessation interventions among those with severe mental illness have suggested the importance of flexibility and choice, a variety of treatment options, facilitators who understand mental health problems and responsivity to the changing needs and preferences of individual service users (29, 30).

EVE, a programme within Ireland's Health Service Executive (HSE) provides a network of services for adults with MHDs. In 2016, the Quit Smoking Programme (QSP) was implemented in 16 of these centers, but the implementation was not evaluated. The current study explores the implementation of this quit smoking programme in the EVE community setting. It is conducted in line with Medical Research Council guidelines (33), and aimed to provide qualitative data, integrating the views and experiences of both service users and facilitators, in relation to this programme's implementation. This study therefore provides richer accounts of the experiences of both staff and service users than that reported in the previous literature, taking full account of the complex issues which can shape the process of implementation in real world mental health settings.

## Materials and Methods

This study adopted an inductive approach, employing flexible interview guides based on the literature and study context but not restricted to fit the domains of a given theory or framework (34).

### Quit Smoking Programme (QSP)

The QSP cessation programme was designed by the HSE Health Promotion Service to provide an accessible resource which sets out a stage-by-stage process to support smokers in the general population in their decision to stop smoking and to sustain the attempt, but was not specifically designed for those with mental health difficulties. Its introduction in EVE services represented the first implementation of this programme within mental health settings and the current study therefore explores its implementation and enablers and barriers to same.

QSP facilitators received a folder detailing the 7-week group programme including questionnaires/forms for attendees to complete regarding their smoking habit, the cost of same, individual quitting plans and personal coping strategies. A carbon monoxide monitor was also provided for attendees to ascertain their current expired CO breath levels. The programme was facilitated by frontline staff who had undergone training in brief interventions for smoking cessation as well as in QSP specifically. Two service users were also trained in QSP and acted as co-facilitators. The programme was introduced alongside a new Tobacco Free Campus Policy.

This study was approved by the Tallaght Hospital/St. James's Hospital Joint Research Ethics Committee on 28th April 2016. All subjects gave written informed consent in accordance with the Declaration of Helsinki.

### Procedures

Recruitment of service user participants was conducted through EVE staff, who once briefed were asked to recruit those who smoke or who had recently quit smoking. A purposive sampling strategy was employed with efforts made to include a range of service users across eligible centers, in terms of age, gender, service use (full-time/part-time) and level of engagement with QSP (35), with a final sample of 20 service users across 9 centers interviewed. Facilitators were recruited by the researcher and an administrative assistant, with efforts made to include all trained facilitators still with the service. Seventeen (of nineteen) facilitators, across 15 centers, participated in focus groups, including both service user co-facilitators.

Service users were asked about smoking; barriers to and enablers of abstinence; feelings on the tobacco free policy and their experience of QSP, with interviews lasting 15–50 min. Facilitators were asked to discuss the recently implemented tobacco free policy; smoking in relation to those with MHDs and health service approach to same; QSP in relation to their training, the resource itself and their facilitation experience as well as initial and ongoing barriers and enablers to starting and running the programme at their centers. In line with the inductive approach taken, guides were reviewed in light of the knowledge gained from the initial round of interviews with minor adaptations made. Focus groups ranged in size from 2 to 6 members and lasted around an hour (49–82 min) with the late arrival of one facilitator to a group managed through an extension add-on interview.

### Analysis

All interviews and focus groups were digitally recorded and transcribed verbatim with identifying data removed and pseudonyms assigned. Data was analyzed using thematic analysis (36) with service user and facilitator data coded separately first prior to integration to produce a coding framework representing key enablers and barriers. Following an extensive familiarization process involving several close readings of transcripts initial codes were generated. Coding of interview transcripts was facilitated using Nvivo10 due to the large number of transcripts while focus group transcripts were coded manually. Once all data had been coded the researcher searched for major themes among these codes and developed an initial framework of themes and sub-themes representing key barriers and enablers. This framework was then discussed with a secondary coder (JS) before AB went on to review and refine these themes to ensure firstly each theme was based on coded data which formed a coherent pattern including exploration of deviant cases where present. Also, secondly and more broadly to ensure the key emergent themes were valid in their reflection of the dataset as a whole (36). A subset of transcripts were read by an additional coder (DR) to confirm that transcripts were coded consistently and that the key findings of the study were supported.

## Results

### Enablers

A number of enablers for, and barriers to, the successful implementation of the smoking cessation programme were identified from the data (see Table 1).

**Table 1:** Enablers for, and barriers to, the successful implementation of the smoking cessation programme.

**Enablers for successful implementation**	**Barriers to successful implementation**
Tobacco free campus policy Resourcefulness of programme facilitators An active, open and engaged recruitment approach Combining with other health initiatives Programme materials and format Health and money as motivators	Implementation of tobacco free campus policy Structure of the service (scheduling, attendance and gaps in availability) and serial nature of programme Lack of a joined-up approach Literacy issues for some Mental health difficulties

#### Tobacco Free Campus Policy

The recently introduced policy emerged as an enabler, leading members to often find smoking more awkward and as a result for some, to find themselves smoking less:

“It has been a little bit awkward so they've cut down…when the weather is bad in particular you'd need to really want your cigarette to go out.” [Facilitator]

The policy also prompted replacements for smoking breaks in some centers with fruit and tea breaks offered to smokers or all members and thus serving as a distraction for smokers.

#### Resourcefulness of Programme Facilitators

The resourcefulness of staff facilitating QSP also emerged as an important enabler for implementation. This was revealed in extra-curricular efforts including the provision of additional individual support where required;

“but I know the person who gave up was struggling hugely when he gave up so I linked in with him every day… just to keep the momentum going and so far he's at around six weeks now off.” [Facilitator]

Facilitators were resourceful in dealing with missed weeks and service users progressing through the programme at different paces. This included meeting more often and building individual work into group sessions to deal with some service users catching up on missed weeks;

“So we kinda go through it and then whether someone's on week 2 or week 3 I'd give them maybe ten minutes and if you're week 2 you get the week 2 little pack and if you're the week 3 and then you do your own little personal bit of writing.” [Facilitator]

Some facilitators used the national quit line and quit website as an additional resource, used technology to support and empower attendees with low literacy, or had members who are former smokers come in to share advice;

“I was just asked and I said I'll just go along…I thought maybe I could give back something” [Service user]

Novel approaches included running a stress management group and linking in with local services:

“In between, rather than kind of doing, you know the same thing all over again the em, just you know the way I did one week about em, you know managing stress and whatever, I just did a relaxation group like.” [Facilitator]

“We got her to give talks and to work with the group as well you know to come in somebody different, we linked up with the local doctors for prescriptions and things like that we linked up with the local chemist as well to provide you know the patches and the different things and the cost and the consequences, so we kinda went out into community with it….yeah and that helped” [Facilitator]

#### An Active, Open and Engaged Recruitment Approach

A more active approach to recruitment seems to have helped get the programme up and running at centers. Facilitators mentioned the need to “sell” the programme a little and to encourage participation:

“Having an initial chat with somebody what you don't want is to just put up a list and say you know ‘Stick your name down there if you wanna do a quit smoking programme alright'…I think this needs to be sold for want of a better word quite rigorously…we are pushing it a little bit and I think if we're not pushing a little bit, we're not doing our job” [Facilitator]

Several service users discussed attending due to curiosity, or just to hear the advice on offer:

“I thought, you know, it's not that I thought about stopping but, em, I thought it's

good to sit down and listen to it and who knows if it gets me down on cigarettes,

that's, why not, you know.” [Service user]

Opening the programme up to those not ready to quit led to greater uptake. And importantly appeared to spur on later quit attempts and personal goals, even if these were an intention to cut-down, but not quit:

“We'd a couple of people who were still smoking coming to the group and no intention of giving up but then, it's like overnight something happens and they come in ‘oh yeah I stopped last week.”' [Facilitator]

In general however, it seemed that while opening up the programme to those not necessarily ready to quit can work, it is best if these attendees have formed meaningful personal goals in advance or are at least somewhat self-motivated to attend. Forced or disengaged attendance may lead to frustration or affect overall atmosphere in the group:

“I'd one who was just like really forcibly letting it be known that the only reason they were there was because people were told they had to be there whatever and …when you're talking about the health benefits and the whatever like and she'd say, ‘[Facilitator Name] I'm just not giving up there's no point in talking to me just I'm only here because people told me so just I don't want to hear any more of it' you know” [Facilitator]

“They asked me to go on it….I'm sitting in on one…. you don't, eh I don't really, you know, spend much time thinking about it” [Service user]

#### Combing With Other and Broader Health Initiatives

Facilitators also mentioned combining smoking cessation with other and broader mental and physical health initiatives and some seemed to feel this could be an effective approach:

“Part of the whole conversation with the Tobacco Free Campus as well as the quit smoking programme is to say you know just to get that message out there consistently to everybody to say we are a HSE location and the HSE are part of the Healthy Ireland programme [a national framework for action to improve the health and well-being of the population of Ireland]…you know many of our centers now are doing healthy eating more often, they're doing mindfulness and quit smoking is just one of those things” [Facilitator]

In some centers service users themselves seemed to naturally start making goals in other areas and link cutting down on smoking with healthier eating or exercise goals:

“[Staff member] in our center is doing the operation transformation with everybody and em some of the people have linked in smoking with that everybody's setting their goals from one week to the next or whatever could be to go for a walk or eat less sugar or whatever but some of them actually putting in to smoke less cigarettes.” [Facilitator]

#### Programme Materials and Format

##### Easy to use, flows, colorful, good information, enjoyable

Facilitators found the pack helpful, with reports that it was easy to use, colorful, flowed well, had good information and was enjoyable:

“What I liked about the document itself…it's you know if I put that down today and I went back and I read this in two months' time, you'd get into it very, very quickly and that's what is, it has to have a nice flow about it” [Facilitator]

Among service users, information and knowledge in relation to the effects of smoking; how to quit; and their own habit emerged as an important aspect of the programme. This knowledge was gained through leaflets and illustrations, teaching and filling in their own information:

“There was very good knowledge. I mean it was about the carbon monoxide, and about your breathing and about the habit and eh there was leaflets about if you give up smoking, em, when you get the craving for a cigarette, how do you distract yourself from doing that, from having a cigarette, you know, do you listen to music, or have a meal or go for a walk, or bring the dog for a walk, or you know…I thought it was a good idea. Yeah it was very good knowing that. It was very good being taught that.” [Service user]

##### Useful tools: questionnaires and carbon monoxide monitoring

Attendees and Facilitators referred specifically to the **questionnaires** that attendees filled in and their usefulness in promoting reflection and revealing habits to the attendee and facilitators;

“There's questionnaires and there's little leaflets you can fill in and then it really points you to how much you smoke and why you smoke, and I think it's really good to understand why you smoke, you know” [Service user]

and also in highlighting the actual cost of their habit:

“The part where you see how much you spend, that was useful, it was a shock to a lot of people how much they actually spent on cigarettes” [Facilitator]

**Carbon monoxide monitoring** in particular, seemed to stand out for service users;

“That really is a wake-up call when you see your levels of nicotine or tar or whatever, em, I find that that's a good help. Then you can see well, you know, this is what you're doing to your lungs or to your body” [Service user]

It was also noted by several facilitators to be particularly useful in raising awareness and reinforcing quit attempts:

“The carbon monoxide, they all loved that, that was a real buzz thing ‘cause it was something real tangible they could actually really see, ok this is what my smoking is doing…there was a real sense of buzz and a few of them really were like, ok knuckling down, after seeing that.” [Facilitator]

There was also however some indication that the carbon monoxide monitor vindicated the e-cigarette for people:

“You get your carbon monoxide levels tested, and mine were the same as a non-smoker.…which I felt vindicated the electric cigarette” [Service user]

##### Doing it as a group–A sense of togetherness

Finally, the communal aspect also emerged as helpful for several attendees;

“It was good to have a group of people that were going through the same thing as yourself, you know.” [Service user]

while facilitators also noted it to be an advantage in attracting attendees, as well as during the programme:

“I love the class I love the interactions in it.” [Facilitator]

#### Health and Money as Motivators for Participants

Physical health appeared to be the main motivation for quitting smoking among service users in general, and an important motivation for joining the programme;

“You know the physical health is suffering, you know, because em I don't have any breath, and I get out of breath. I get very tired, I get very tired and I get breathless very quickly and very easily you know…that's what's motivating me to go on the programme, and try and see if I can do something about it.” [Service user]

“Health is the big one, health is the biggest motivator, even over the financial one” [Facilitator]

Overall while health was the primary motivator, money also emerged, but was usually secondary;

“and then definitely financial it adds to it as in somebody has more money then to go and treat themselves or to do something nice or to put it away or whatever.” [Facilitator]

“It's bad for your health first of all and secondly it's, it's money that I could use somewhere else.” [Service user]

though notably for some, the financial aspect was in fact primary:

“Eh money (Laughs), quit smoking have more money in my wallet. I wasn't too concerned with my health, eh but now I am so” [Service user]

### Barriers

#### Implementation of Tobacco Free Campus Policy

##### Ongoing facilitation of smoking

Evidence of differential tobacco free campus policy implementation across settings pointed toward the ongoing facilitation of smoking at some centers. Smoking location did not appear to have been affected at all in one center, in another a service user described the erection of a smoking shelter in a new area, while at another there was the nomination of an alternative sheltered smoking spot for wet days.

The culture of the center also seemed to impact on the Tobacco Free Campus Policy. At Center Q the culture meant that the policy involved only a change in location and there were no limits to when service users could smoke;

“People leave our gates, walk across the road and there's a big congregation of people there, em almost constantly…there's a lot of people who aren't engaged you know and em, they just come in and out constantly you know and that's the way the center was historically, like it's very kind of family orientated environment very easy going and we don't tend to kind of do parent child stuff.” [Facilitator]

The ongoing facilitation of smoking at some centers, which in some cases was due to having a shared campus, was, it seems, in some casesa barrier to service users trying to cut down or quit smoking.

##### Associated forced/‘herded' attendance at QSP

At Center F, the ongoing facilitation of smoking was more apparent as Tobacco Free Campus Policy was not implemented due to feasibility issues relating to location and traffic. Instead, a derogation was granted, with the trade-off that attendance at QSP was compulsory for all smokers:

“Well there was a derogation in our center, em it was an issue, going out onto the road would be dangerous like with that but it's also very far away but em, we'd a derogation that we could still use the smoking shelter em, if on the condition that everybody did the programme.” [Facilitator]

While attendance was not compulsory at any other center, others also felt that the timing in relation to QSP following the policy may have had a negative effect on morale:

“When the ban came in it was like the two were at the one go you're trying to force me to stop smoking and you're trying to make me do this course to get me off…yeah it was kinda just because the two of them did happen at the same time …just gave it a negative slant.” [Facilitator]

#### Structure of Service (Scheduling, Attendance, Gaps in Availability) and Serial Process Assumed

##### Scheduling

Scheduling barriers emerged around finding the right start date and day for the programme as service users do not all attend 5 days a week:

“There's people attending 3 days, and there's people attending 2 days, and there's people attending 4 days and whatever and to try and find the spot where you were trying to get as many like, I changed the timetable recently…there was only 3 people the last week…I'll have to look at the timetable again but it's very hard.” [Facilitator]

Facilitators also commented on the clubhouse structure (which permits flexible attendance) and several felt that this in particular meant it was difficult to know when to start a programme, as attendance can be irregular in general.

##### Contractual obligations

Contractual obligations also meant smoking cessation groups had to be run in the afternoon when attendance is lower. In general smoking cessation was less of a priority meaning that it was also the “first thing that will go”:

“That was the biggest thing for ourselves, it was just em, although it's part of policy and now the actual quitting smoking, but facilitating it is the first thing that will, I'll be honest in our center, if something's to go in the timetable, it'll be the first thing that will go.” [Facilitator]

##### Staff sickness, leave or moves leading to gaps in availability

Staff shortages, sickness and other leave were often the reasons something had “to go” in the timetable and facilitators discussed how sickness or leave often meant the programme simply did not happen in their absence. This was also observed by service users;

“There was a few stops in it. One of the, the person that was giving it mightn't be in that day or on holidays or sick or something.” [Service user]

##### Serial/logical process

Some facilitators felt the 7-week programme assumed a very swift, logical and serial process that was perhaps unrealistic. In reality, service users were not always ready to quit, or even to set a quit date on the designated week:

“Yeah, we didn't get past week 3. I would say yeah no we didn't em, again so we get, so we started first we had a good first week if you like, and the second week was fine and then the third week we'd no-one setting dates” [Facilitator]

“They want you to stop after seven weeks. I wouldn't be able to” [Service user]

Facilitators were sometimes left feeling stuck and unsure what to do. There was a seeming lack of clarity on how to move forward when these issues arose in this real world setting:

“It would be helpful within this [pack] or whether EVE kind of have an addendum to this saying what to do if you if this scenario happens, you get stuck at week 3.” [Facilitator]

While some felt they had to stop, other facilitators made the decision to keep going. Some centers also made efforts to tailor the programme so that it was at individuals' own pace, and this together with decisions by some service users to re-attend a second or even third time, illustrates the longer more circular process quitting could be for some service users:

#### Inconsistencies and the Need for a Joined-Up Approach

##### Lack of healthcare professional (HCP) advice

Smoking cessation did not emerge as strongly addressed by healthcare professionals. Several service users and staff reported a complete lack of smoking cessation advice from HCPs:

Facilitator 1: I did specifically ask one of my keyworkers about that, he'd been to his nurse for his injection and ah…I just said aw did your nurse ever mention your smoking or does your psychiatrist you know, he only sees him every 2 or 3 months now, no no it's never mentioned…

Facilitator 2: They never mention it

This was also supported by several service user interviews where service users commented in relation to HCP advice that “It never happened.” The exception to this was two service users who reported in their interviews that their GP had continually raised smoking as a health issue.

##### Inadequate HCP Advice

Some of the interactions around smoking that did occur were revealed to be ill-informed in relation to inaccurate advice as recounted by one facilitator;

“Some were told by their doctors and I know this is wrong information that they couldn't use any of the smoking, giving up smoking aids because it would affect their medication.” [Facilitator]

or ill-advised approaches in terms of a seeming focus on restriction of access to cigarettes rather than offer of person-centered support to quit:

“Actually one of the last times I was in [specialized neuro-psychiatric hospital], just to be noted, they said we're going to have to take your e-cigarette off you, I said why? We're putting everyone on the patches” [Service user]

Several service users reported bringing up smoking and Nicotine Replacement Therapy (NRT) themselves;

“Yeah, no he [GP] didn't suggest it [patches]. No I did” [Service user]

while many HCPs seemed to avoid actual recommendations of quitting. These ranged from vague almost neutral references to smoking, to advice to cut-down rather than quit, to advice that they did not need to quit;

“I always said I'd give them up if the doctor told me to…But he never did. He'd say, ‘You're still smoking”' [Service user]

“I go to a psychiatrist and I told him that I'm trying to give up the cigarettes, and he told me to cut down. [Service user]

“Yeah my doctor discussed it one day and he asked me and I said, I have a few, andhe said that's no harm, he said…He said don't mind, have a few if you want to yeah.”[Service user]

##### Lack of a joined-up approach to cessation support

There were calls for a more joined-up approach;

“If everybody does a little bit and they're all going in the same direction, they don't all have to be singing on the same page, but if everybody's going the same direction, you're planting seeds as you go along.” [Facilitator]

and the need for smoking cessation support to be brought into hostels and hospitals:

“Help with quitting. Not just shut down completely like. I mean just introducing as much as they can into hospitals and there might be like half the patients might want to quit, half of them might not want to and at least give people a chance, you know.” [Service user]

Facilitator 2 used the example of the person-centered plan, which is made for each service user at meetings including doctors, nurses and hostel staff and queried why smoking could not be addressed here when weight already is;

Facilitator 2:When you're doing a person-centered plan for someone and the hostel staff are there, the doctor's there, or the CPN is there or whoever, that the health, if a person had weight issues that would be to the fore at every single meeting…but if we do that for weight, well why not do that for smoking [agreement], it's the same thing d'you know so you should be, it should be, like I think smoking and if if it, it is affecting a person's health, you can't say if it is affecting the health, it should be in that PCP.

while Facilitator 1 noted that this joined-up approach should be at policy level as given under-resourcing and lack of staff services are unlikely to “opt in.” The lack of a current joined-up approach also meant that for service users due to leave EVE the availability of continued smoking cessation support is a gray area, an uncertainty which emerged in several service user interviews.

##### Exemptions and inconsistencies in relation to tobacco free policies

In addition to a frequent lack of advice and joined-up cessation support, there were also inconsistencies in relation to smoking policies in hostels and psychiatric hospital settings;

“I was in a smoke free hospital…but [the] psychiatric unit had their own yard…there's a smoking shed” [Service user]

and the maintenance of smoking as a social activity in these settings seems to lead to service users increasing consumption and even relapsing to smoking at times:

“If she went out for a cigarette she'd have someone to talk to outside, so she found when she came out of [acute mental health unit] and into here, she was after increasing about 20 on top of what she was smoking.” [Facilitator]

“I was meeting loads of new people and I felt oh God I can't, I need something to take the edge off, so I started smoking again, because everyone was out there talking and chatting and I was saying aw me sitting in here. I want to be out with them, you know…yeah, I went back on them.” [Service user]

##### Facilitators need to be linked

Within EVE itself, the lack of a linked-up approach to QSP was a barrier, as an opportunity for facilitators to collaborate and share knowledge was lacking.

“Maybe the organization would be happy enough to, to okay us to meet up, once or twice a year to kind of get together and share experiences and share feedback…just give each other a call and the knowledge is there (Facilitator).

#### Literacy Issues for Some Participants and Need to Add Technology Component in General

Participant literacy was an issue for some facilitators and could cause difficulties with engagement and pacing, while Facilitator 8 found the questionnaires involved and group format meant she felt she was effectively revealing any literacy issues:

“One of my problems as well…I'd two people that couldn't read, write or spell and that was very difficult now I have to say, because I was more or less filling it out for them and I don't know if I felt uncomfortable, because you know it was sort of em, you were letting the rest of the group know that they couldn't read or spell and that you know, I just felt it was awkward.” [Facilitator]

##### Technology could help

There was suggestion from several that additional use of technology could not only help with literacy issues but also overcome issues like missing questionnaires. This might lead to enhanced ownership with service users even able to log in at home. In relation to centers, the point was made that many of the resources in terms of computers and equipment are already in place.

#### MHDs

An explicit association between MHDs and smoking emerged among service users, in relation to both their own and others' smoking:

It was reported as a specific reason for starting;

“I became unwell [developed psychosis and depression], em, and I went on medication and I started smoking regularly then every day.” [Service user]

for relapsing; and at times described as the reason for becoming a smoker despite wishes not to be, seemingly taking the choice out of their hands somewhat.

Although a few facilitators felt that quitting smoking was no different for those with MHDs;

“I just think they're the same as everybody else, I don't think because of the mental health difficulties it's any harder to, to quit.” [Facilitator]

many felt that it can indeed be more difficult for this group.

A number of factors were felt to contribute to this:

##### Coping mechanism

Smoking emerged strongly as a coping mechanism from the perspectives of both facilitators and service users themselves.

Coping mechanism for stress

Its role as a coping mechanism for stress was evidenced when initiation, increases and relapses were described in the context of acute stressors in the past. These acute stressors included health scares, family members falling ill and bereavements:

“The first time ever, because my father actually gave me bad news at the time. My mother was in hospital and she was in a bad way in hospital.” [Service user 1 on starting smoking]

In addition to acute stressors, smoking was also used to cope with the everyday stresses of life:

“What doesn't help me quit smoking, em, day-to-day living, you know, being caught in traffic jams, or you know, being short changed in a shop or dogs barking at me, or if it's gale-force winds and pouring rain, and I'm after forgetting my umbrella, and you know, things like that. Just day-to-day living, you know, that would make it difficult to stop smoking” [Service user]

Coping mechanism for MHDs

Its role as a coping mechanism also emerged specifically in relation to MHDs such as anxiety;

“If I'm anyway anxious I, I tend to unfortunately, and I used to be real confident and I'm losing my confidence a bit again, em, but if I'm worried about anything, (whispers) oh God I need a cigarette, and then I'd be grand for a while, you know” [Service user]

and schizophrenia or psychosis;

“[smoke] to keep me calm…well [pause] there's mood changes. People with schizophrenia have mood, mood changes, and em, eh a cigarette calms it down a little bit. You'd, you'd enjoy because it just calms you down, you know.” [Service user]

“I've psychosis…it [smoking] makes me feel easy, and em it kind of [pause] it kind of relaxes me that bit” [Service user]

with a dose response relationship again seemingly emerging for some;

“She said like, when I'm very unwell I smoke extra” [Facilitator]

“I probably would have become more anxious in the last few years…so I think that would be the cause of [becoming a heavier smoker]” [Service user]

Effectiveness as a coping mechanism

While many service users strongly believed in smoking's effectiveness in relieving and calming stress and symptoms related to MHDs;

“They help to relieve stress” [Service user]

some facilitators and service users expressed doubts regarding its effectiveness as a coping mechanism;

“You know it doesn't help, it's never, there's no actual evidence to say it supports em, your stress levels” [Facilitator]

“I don't think it makes a difference” [Service user 11 (who here contradicted her earlier report that smoking helps with her depression)]

and thought rather than help it could be an added source of stress or anxiety;

“I don't believe that em, smoking, even having smoked, I think you can delude yourself that it does em, when you're smoking, I don't believe that smoking helps you with stress…if I'd no cigarettes…then you have a stress on top of the stress” [Facilitator]

“Of more recent times, the smoking causes my anxiety…because it makes me think I'm going to get cancer, or I'm going to take a heart attack, or I'm going to take a brain hemorrhage or something.”[Service user]

and overall, even if effective, the trade off in terms of physical health perhaps outweighs any benefit:

“I just think it's a general mind-set change of saying, you know, the easy thing to do is say ‘Look carry on smoking because otherwise you're going to be mentally distressed' or whatever it is and you know there might be an element of truth to that, but longer term you're going to, you know one in two of them are going to die” [Facilitator]

“They help to relieve stress, you know…yeah, de-stress but I found you…I'd say that ah overall you know, that they probably weren't, they're probably having a bit of a negative effect on me…my overall health would have, would have changed…you know, I wasn't as physically active as I was a couple of years ago you know. So, I suppose, the smoking doesn't help that, you know. It could help to, to solve stress a bit, you know, but physically it probably isn't such a great stress helper.” [Service user]

Perceived as “Need”

In a couple of cases, the notion that smoking is a coping mechanism or crutch for this group went a step further and it was labeled a “need.”

“I need it for my mental health” [Service user]

This occurred at service user and family levels and actually led to a relapse to smoking for Service user 2:

“My mother knew I was bored after my dad died, and she knew I, she thought I needed something so she bought me cigarettes” [Service user]

##### Lacking self-belief/self-esteem

Another barrier which emerged among both facilitators and service users, related to a lack of self-esteem or self-belief among service users. Several facilitators described this lack of self-belief;

“Some people's self-esteem and their belief in themselves wouldn't be at a high point, so you'd be trying to encourage and that they can you know, so that that might be a barrier with some of the mental health.” [Facilitator]

with a lack of perceived ability also emerging in some service user interviews:

“I knew deep down that I wouldn't be able to…will I try? Will I be able to try?” [Service user]

For others a lack of self-belief did not emerge and two service users were in fact particularly clear on their ability to quit, but simply not ready at the moment. In both cases, this self-belief seemed to be the result of previous quitting.

##### Lack of consistent determination/willpower

There was some suggestion from facilitators and service users that those with MHDs experience a barrier to quitting in the form of an associated lack of determination or consistent motivation:

“You have to be motivated and motivation is always a kind of a critical factor as well and while the, person may be motivated one week, you know 2 or 3 weeks in they may not feel so good and then that's their downfall then that it falls, it falls you know, it doesn't necessarily work for them” [Facilitator]

While not an issue for Service user 12, he again seemed to associate mental illness more generally with decreased strength or determination:

“I am a very strong minded person even though I have mental illness and all…I'm very strong minded in the sense if I say I'm starting Monday, no smoking. I will start Monday and there'll be no smoking.” [Service user]

In general, he seemed to need the assistance of the programme less with a clear focus on his own plan and preparation. This contrasted with Service users 18 and 14 whose reports suggested they wanted or needed more external pressure to quit or set a date:

“People like that, facilitators need to give me a good boot in the backside, and say, “[Name] get in there and buy them patches.” [Service user]

“I would have like that they pinpoint a bit more out on stop the smoking. It's to stop smoking and they don't do that either there, you know, they are very careful with not to say that [you have to stop smoking], you know…and I find that amazing that they don't, even a cessation group, they don't do that either, you know. I think I need that. I think I need that somebody says to me you stop smoking now.” [Service user]

Service user 14 did go on to state however, that she would prefer to be given a quit date in the future rather than told to stop straight away:

“I'd rather have a date in the future so that I can get used to it, you know” [Service user]

##### Smoking among peers and past culture

The prevalence of smoking among people with MHDs may also represent a barrier given the culture of smoking this creates among peers.

There was also some evidence of this increased prevalence and a culture of smoking at EVE centers:

“You get shanghaied into going out for a cigarette…ah no there's, there's one or two that smoke here and if they go outside, they'll give you a cigarette and you have one.” [Service user]

Centers also experience issues such as a culture of money lending and borrowing to buy cigarettes;

“One other thing, em, came up there, just thinking em, people who smoke like that financially they don't have as much money, so that, there was a knock on effect so they wouldn't have as much money and like [Facilitator 2] said they'd do anything to get a cigarette so borrowing became an issue… and as a result there's problems then people are not giving back the monies so there was huge knock on effects from that.” [Facilitator]

as well as trading of counterfeit cigarettes;

“and then the dodgy ones as well, a guy going in [to town] buying them, and I've seen it in centers, coming in on Monday with like 200 cigarettes and doling them out.” [Facilitator]

Other reports however contradicted this and overall there was a sense the culture of smoking was waning:

“Smoking has kind of died down here.” [Service user]

“I think it was a lot higher when I first started. This is even well before smoking cessation and now it's, I'd say we're twenty, twenty percent maybe smoking twenty-five maybe.” [Facilitator]

Facilitators commented on the lower prevalence among younger service users;

“There was a culture of go and have a cigarette I think going back but that's definitely not the case with the younger people coming in. I don't think we have as many smokers definitely with the younger em variety.” [Facilitator]

while for some the past culture of smoking also seemed to serve as an extra motivator to provide support now:

“We have a responsibility to address this I believe, because our services have condoned this for so long and the medical community probably exacerbated it over the years, made it worse so we have a responsibility to kind of, maybe be a little more of campaigners about it.” [Facilitator]

##### Lifestyle: lack of structure/activities

Inactivity, lack of structure and time spent alone emerged as particular barriers for service users from both facilitators and service user perspectives. Service users discussed inactivity, boredom, time alone and filling a vacuum as prompts to smoke:

Interviewer: And if I was to ask you why did you smoke, what would you say?

Service user 7: Em, I'd say the vacuum after I got sick, and being off work, having that time on my hands.

This was mirrored in the accounts of several facilitators, who also noted a lack of structure and time alone as key barriers for individuals with MHDs.

Hobbies, work, and being otherwise busy often emerged as distractions;

“There are places I smoke less. Yeah. When I'm doing something I get it done, you know” [Service user]

Service user 15 referred to the years when he stopped smoking as partially down to being busy with work;

“Well I was working away like and there was no point to be smoking while you were working, you know, I'd be stopping every 5 min if I had of been smoking at the time, so it helped me as regards work as well.” [Service user]

while Service user 12 described how his hobby serves as a powerful distraction;

“I smoke an awful lot less when I'm gigging” [Service user]

There were calls from service users and facilitators for more activities;

“I think someone mentioned it, just in terms of structure I think structure is, if a person's day can be filled and it doesn't matter whether it's you know within the center, outside the center, in terms of leisure activities and social activities, that's obviously going to help in in a big way” [Facilitator]

and some felt there was a need in particular for specific replacement activities though concrete suggestions were limited:

“What to do when they do give up cigarettes. I found like, I started doing myself knitting and reading more and doing things like that, I think something like that could be put into the book, what to do, [murmuring] ideas and suggestions…I feel what we need to do is encourage people to change their lifestyle, I think that would help an awful lot.” [Facilitator and Former Smoker]

“Something to do with their hands, you know, knitting or sewing or something like that…but my, my hands shake so it's very difficult for me to, to do things.” [Service user]

##### MHDs as an excuse rather than an actual barrier

There was also some suggestion from facilitators that in some cases, MHDs can act as an excuse at the individual level rather than a barrier in terms of actual ability to quit smoking. This is perhaps unsurprising given the past culture discussed above.

“I think there can be some people who might use their, their mental health as an excuse…they might be in a system where they might have a nurse or a key worker who is facilitating and done a lot for them and now they're signed up to a smoking cessation programme but now it's them that has to do it, nobody else is going to quit for them.” [Facilitator]

Facilitator 7 addressed this by highlighting the equality between service users and staff;

“I've used me as an example in my groups ‘So you're different to me because you have a MHD? Nah you're no different to me you're able to do loads of things I can't do…so if you I think if we hit it at that level and then all of a sudden they're looking at you and they start laughing, they know straight away, they're like alright I'm not gonna use that.” [Facilitator]

which is also central to the structure and ethos of the service more generally.

##### Timing

While many felt MHDs can indeed make quitting harder facilitators felt timing can be a really important factor as a barrier to attending the programme at all;

“The interesting thing about the first session was that the person, it was Christmas time it was the wrong time and they found by, I think we got to week 5, they found that week 5 they had to stop because their cigarette smoking had increased! Because of Christmas and stresses and all that kinda thing and all so” [Facilitator]

but also to actual quitting in those that did attend:

“I did the course 3 times in [name of location] and she sat on each one of them, but she had something going on in her life that she just wasn't ready and…it was a bit her mental health was a bit unstable at the time, yeah and she had family issues she had to get sorted out and she just felt she needed that cigarette.” [Facilitator].

Table 2 summarizes the sub-theme MHD barriers discussed in this paper providing further sample quotes.

**Table 2:** Summary of barriers related to mental health difficulties with sample quotes from service users and facilitators.

**MHD barrier**	**Sample quote**
Perceived association between MHDs and smoking	“There was eight of us in the family, and I'm the only smoker and I put it down to my anxiety” [Service user]
	“When I became a teenager I said to myself I'm not going to ever smoke, and I'm not, I want to be fit and keep myself fit and healthy, you know, and em, it didn't happen that way, with the illness [schizophrenia] I had, it just didn't materialize like that.” [Service user]
	“There's no doubt it is, it is far more difficult for them to give it up.” [Facilitator]
Coping mechanism	“It's a safety net, isn't it yeah” [Facilitator]
	“Like the cigarettes are just such a big crutch” [Facilitator]
	“It helps me calm down…it helps, em, I'd have a cigarette when I'm stressed or anxious, and I'd have a cigarette and I'd feel grand then for a long, for a few hours…it makes me feel better…it helps me relax. It helps me to, you know, get my bearings sometimes.” [Service user]
Lacking self-belief/ self-esteem	“And some of them again when they say I don't want to give up or I'm happy smoking they might even be saying, do you know what I'd love to give up, but I'm just not able to, you know I'm not able to do that.” [Facilitator]
Lack of consistent determination/willpower	“It's not having the willpower to stop doing something that's very bad for you…if you have a mental health problem it's harder to give up smoking because I just haven't got the mind, I haven't got the willpower and I'm not able to make a decision to say I'm going to quit, and then just quit. I can't, I find that difficult to do.” [Service user]
Smoking among peers and past culture	“Just about everybody I know in the psychiatric services, service users, people with mental health, people with mental illness, they all smoke.” [Service user]
	“Here in the clubhouse I have contact with smokers, and we all go together for our smoke, if we can, especially the women, you know, who smoke and they get it together. That's in a way nice. It's chatty you know. Yeah.” [Service user]
Lifestyle: lack of structure/alternative activities	“A lot of it was to do with like boredom” [Service user]
	“They actually said it to me…yeah when I'm in the flat on my own at the weekends, that's when I tend to smoke a lot.” [Facilitator]
MHDs as an excuse rather than an actual barrier?	“Em I think depending on the groups sometimes the ones with the mental health difficulties will use it as a tool to not move forward and they'll kind it use it as a as you know, an excuse almost” [Facilitator]
Timing	“What he's going through at the minute, there's no way that he's ready or there's no way he wants to yet but that's not saying down the line” [Facilitator]

## Discussion

This study aimed to identify key enablers of, and barriers to, the implementation of a quit smoking programme in community adult mental health services. By exploring an ongoing group smoking cessation intervention in a real world mental health setting, from both facilitator and service user experiences of its implementation, this study filled an important gap in knowledge.

The emergence of health and money as important motivators is consistent with previous research in relation to the enablers of smoking cessation among individuals with severe mental illness (37, 38), and is similar to other populations (39–41). The use of carbon monoxide monitors makes use of biofeedback, which is arguably one of the strongest behavior change techniques (42), and appeared to be a powerful, and appropriate, aspect of this programme also, spurring cessation efforts as well as reinforcing those already quit. Interestingly however the monitor also appeared to vindicate the use of e-cigarettes in service user's eyes, presumably an unintended consequence given the HSE have not endorsed e-cigarettes as a cessation aid (43).

The emergence of facilitator resourcefulness and tailoring of the programme to individuals' needs as an enabler is consistent with previous studies among mental health patients. These studies found flexible, personalized and responsive approaches to be helpful with benefit emphasized by both facilitators and service users (30, 44). Notably, this flexible, individualized approach is also consistent with the person-centered philosophy underpinning delivery of EVE programmes in general. An active, open and engaged recruitment approach seemed to work best as opening up to those not ready to quit, but interested in attending, sometimes led to unexpected wins by spurring on later quit attempts. Non-voluntary attendance without personal goals however appeared to lead to disengagement, frustration for both service users and facilitators and potentially affected overall group atmosphere.

The recently introduced Tobacco Free Campus Policy emerged as both an enabler and barrier to programme implementation and participant quitting. The identification of the partial nature of the ban and its varying implementation as barriers supports other studies which show partial smoke-free policies are less successful than total smoke-free policies. A partial ban creates additional problems and has a limited impact on the staff and service user culture of smoking (45). Moreover, the timing of implementing cessation support, following or alongside the introduction of a smoke-free campus policy, may have negatively affected morale.

Conflicting priorities and a lack of prioritizing smoking cessation support as part of staff workload has previously emerged as a barrier among HCPs in Ireland and internationally (46–48). As well as in relation to mental health services more specifically especially in the context of outpatient settings (38).

The emergence of the current lack of a joined-up approach as a barrier producing inconsistencies in relation to lack of cessation supports and also in relation to exemptions from tobacco free policies echoed the conclusions of a recent review of qualitative studies which called for cessation to be addressed at all levels (systemic, health provider, and individual) among people with severe mental illness (37). Although there is a lack of evidence relating to the effectiveness of advice for those with serious or severe mental illness (49, 50), the motivational impact of HCP advice, where it did occur, was important. This is not surprising, given prompts from health professionals have been shown to be an important driver in quit attempts among smokers in general (51–53).

In line with previous research, barriers relating to MHDs including smoking as a coping mechanism (44, 54–57); lack of self-belief (29, 54, 56); lack of consistent motivation (30, 38, 44); prevalence of smoking among peers and the culture within mental health settings (37, 44, 58); and lack of structure or alternative activities (59–61), also emerged from the data. Timing was also perceived to be an important factor by facilitators and some service users felt an inpatient stay was the wrong time for cessation. Evidence is still however lacking in this area due to the ongoing tendency for smoking cessation studies to recruit from psychiatrically stable rather than acutely unwell populations (50, 62).

### Implications

There was some evidence in the current study, of knowledge among service users and facilitators of the ineffectiveness of smoking as a coping mechanism and the ability of those with MHDs to quit. Overall, however, findings suggest that integrating education on the proven ability of people with MHDs to quit smoking, and the benefits of quitting for mental health, into facilitator training and resources for service users could be beneficial. It is also crucial that the service continue to present this opportunity to quit on an ongoing basis, especially given the accounts of non-attenders who also expressed the wish that this service remain as they may use it in future.

A stronger focus on replacement coping mechanisms and activities may be needed. It is important however to ensure these replacements are healthy. Given the increased physical health risks in general seen among those with MHDs (4, 63), the introduction of well-intentioned replacements such as biscuit breaks seem ill-advised. Rather, a focus on overall wellness and combining smoking cessation with broader health initiatives, a proven enabler, should continue; as should the open recruitment of those not ready to quit but interested in attending and perhaps cutting down. In light of the potential changing levels of motivation among those with MHDs, which were also reported in the current study, Williams and Ziedonis have previously recommended reduction toward abstinence as a method of harm reduction (64). Smoking cessation support should also ideally be in place in advance of the introduction of any new smoke-free policies. Appears that the introduction of a smoke-free policy alone is insufficient. Appropriate implementation by enthusiastic staff with environmental barriers addressed in advance is required.

Finally, attending the cessation programme and attempting to quit turned out to be a longer and more circular process than anticipated for some, providing support for the idea that some individuals with MHDs may require more intensive, modified or tailored cessation support (25, 37, 65). It is important to note however that some service users in the current study needed little support to quit particularly in the early stages of implementation. Perhaps the introduction of new smoking policies and a support programme produce quick initial gains or “low-hanging fruit” in terms of the quitting of more motivated and less dependent smokers after which those more dependent smokers, needing greater support, remain. Regardless, there was a clear need to sufficiently equip facilitators, through enhanced training, guidelines or a nominated support contact, for attendees or groups getting “stuck” in order to avoid feelings of uncertainty and frustration. Beyond a support person, a forum for facilitator communication and collaboration could also be beneficial in sharing knowledge and approaches to obstacles.

Future research may wish to explore how the provision of evidence of successful smoking cessation in those with MHDs, as well as a greater focus on replacement coping mechanisms and activities, might enhance a smoking cessation programme for this population. Studies which include exploration of experience of use at both participant and provider levels in addition to cessation outcomes would be particularly useful.

### Strengths and Limitations

This study was strengthened by the participation of a high number of service users across a large number of the eligible centers as well as the inclusion of almost all facilitators, meaning results should have good external validity. Including the 16 sites meant contextual factors were well accounted for and barriers and enablers that emerged were truly cross-site. Previous qualitative studies of smoking cessation and other lifestyle interventions in individuals with serious or severe mental illness have at times involved very small samples of service users (66, 67), while others have failed to include the service user voice at all (38, 44).

Beyond the inclusion of both voices, the triangulation of sources, combining both service user and staff perspectives and experiences, also serves to validate study findings (68), adding credibility and strengthening confidence in the conclusions drawn (69). Lambert et al. have also found that the integration of focus group and interview data in particular, assists in the identification of individual and contextual circumstances, thus adding to interpretation and ultimately enhancing trustworthiness of results (70). The two-phase sequential design also allowed for the refinement of the facilitator focus group interview guide and thus allowed service users to set the agenda somewhat before facilitator data collection commenced. Unfortunately, due to incomplete data collection by staff, quantitative data on programme outcomes were unavailable so we were unable to include a mixed methods triangulation element to the data.

As this was a qualitative study, findings are not generalizable beyond the study population and conclusions drawn refer to the sample itself (71). Practical issues around staff availability led to pragmatic decisions including conducting a focus group with just two members and allowing a facilitator to join another focus group late. The recruitment of participants through HSE staff may have meant they did not believe the researcher was truly neutral and interview data confidential, although this was restated at the beginning of each interview and focus group, it is unclear if this affected findings.

## Conclusions

A group-based smoking cessation programme with an open recruitment approach and the provision of individual support, where needed, appeared to work well in community mental health services. Findings indicated that implementation of cessation programmes in community mental health settings may be best when done in advance of new tobacco free policies, when it is prioritized and sustained and when the cessation care provided also addresses the key barriers perceived as specific to those with mental health difficulties. More broadly, a joined-up approach across the health service seems necessary to address ongoing inconsistencies and support those with MHDs in their efforts to quit.

## Author Contributions

AB designed the research instruments, completed all data entry and data analysis and wrote the manuscript as part of her PhD. JS provided feedback on research instruments, supervised AB in the conduction of thematic analysis and assisted in review of the current paper. LC provided feedback on research instruments, supervised AB in completing the study and assisted in review of the current paper. MW, GS, and TO'B provided original research ideas, designed the protocol and applied for research ethics approval, contributed to the design of research instruments and assisted in review of the current paper. DR contributed to the data analysis of the manuscript, and assisted in review of the current paper. FD was the principal investigator for this project. He supervised the project, provided feedback on the design of research instruments, qualitative analysis and assisted in review of the current paper. All authors revised the original manuscript and provided critical input.

### Conflict of Interest Statement

The authors declare that the research was conducted in the absence of any commercial or financial relationships that could be construed as a potential conflict of interest.

## References

[1] Lasser K Boyd JW Woolhandler S Himmelstein DU McCormick D Bor DH. Smoking and mental illness: a population-based prevalence study. JAMA (2000) 284:2606–10. 10.1001/jama.284.20.2606 11086367

[2] Royal College of Physicians Royal College of Psychiatrists. Smoking and Mental Health. London: RCP. Royal College of Psychiatrists Council Report CR178 (2013).

[3] Lawrence D Mitrou F Zubrick SR. Smoking and mental illness: results from population surveys in Australia and the United States. BMC Public Health (2009) 9:285. 10.1186/1471-2458-9-285 19664203 PMC2734850

[4] Burns A Strawbridge JD Clancy L Doyle F. Exploring smoking, mental health and smoking-related disease in a nationally representative sample of older adults in Ireland – A retrospective secondary analysis. J Psychosomatic Res. (2017) 98:78–86. 10.1016/j.jpsychores.2017.05.005 28554376

[5] Sokal J Messias E Dickerson FB Kreyenbuhl J Brown CH Goldberg RW. Comorbidity of medical illnesses among adults with serious mental illness who are receiving community psychiatric services. J Nerv Ment Dis. (2004) 192:421–7. 10.1097/01.nmd.0000130135.78017.96 15167405

[6] Carney CP Jones L Woolson RF. Medical comorbidity in women and men with schizophrenia: a population-based controlled study. J Gen Intern Med. (2006) 21:1133–7. 10.1111/j.1525-1497.2006.00563.x 17026726 PMC1831667

[7] Batki SL Meszaros ZS Strutynski K Dimmock JA Leontieva L Ploutz-Snyder R. Medical comorbidity in patients with schizophrenia and alcohol dependence. Schizophr Res. (2009) 107:139–46. 10.1016/j.schres.2008.10.016 19022627 PMC2649875

[8] Copeland LA Mortensen EM Zeber JE Pugh MJ Restrepo MI Dalack GW. Pulmonary disease among inpatient decedents: impact of schizophrenia. Prog Neuropsychopharmacol Biol Psychiatry (2007) 31:720–6. 10.1016/j.pnpbp.2007.01.008 17292522

[9] Himelhoch S Lehman A Kreyenbuhl J Daumit G Brown C Dixon L. Prevalence of chronic obstructive pulmonary disease among those with serious mental illness. Prevalence (2004) 161:2317–9. 10.1176/appi.ajp.161.12.2317 15569908

[10] Partti K Vasankari T Kanervisto M Perälä J Saarni SI Jousilahti P. Lung Function and Respiratory Diseases in People with Psychosis: Population-based study. Br J Psychiatry (2015) 207:37–45. 10.1192/bjp.bp.113.141937 25858177

[11] Jones DR Macias C Barreira PJ Fisher WH Hargreaves WA Harding CM. Prevalence, severity, and co-occurrence of chronic physical health problems of persons with serious mental illness. Psychiatr Serv. (2004) 55:1250–7. 10.1176/appi.ps.55.11.1250 15534013 PMC2759895

[12] Callaghan RC Veldhuizen S Jeysingh T Orlan C Graham C Kakouris G Patterns of tobacco-related mortality among individuals diagnosed with schizophrenia, bipolar disorder, or depression. J Psychiatr Res. (2014) 48:102–10. 10.1016/j.jpsychires.2013.09.014 24139811

[13] Tran E Rouillon F Loze JY Casadebaig F Philippe A Vitry F. Cancer mortality in patients with schizophrenia: an 11-year prospective cohort study. Cancer (2009) 115:3555–62. 10.1002/cncr.24383 19548261

[14] Lawrence D Hancock KJ Kisely S. The gap in life expectancy from preventable physical illness in psychiatric patients in Western Australia: retrospective analysis of population based registers. BMJ (2013) 346:f2539. 10.1136/bmj.f2539 23694688 PMC3660620

[15] Ho SY Alnashri N Rohde D Murphy P Doyle F. Systematic review and meta-analysis of the impact of depression on subsequent smoking cessation in patients with chronic respiratory conditions. Gen Hosp Psychiatry (2015) 37:399–407. 10.1016/j.genhosppsych.2015.05.002 26022383

[16] Doyle F Rohde D Rutkowska A Morgan K Cousins G McGee H. Systematic review and meta-analysis of the impact of depression on subsequent smoking cessation in patients with coronary heart disease: 1990 to 2013. Psychosom Med. (2014) 76:44–57. 10.1097/PSY.0000000000000020 24367125

[17] Taylor G McNeill A Girling A Farley A Lindson-Hawley N Aveyard P. Change in mental health after smoking cessation: systematic review and meta-analysis. BMJ (2014) 348:g1151. 10.1136/bmj.g1151 24524926 PMC3923980

[18] Meernik C McCullough A Ranney L Walsh B Goldstein AO. Evaluation of community-based cessation programs: how do smokers with behavioral health conditions fare? Commun Mental Health J. (2017) 54:158–65. 10.1007/s10597-017-0155-2 28770359

[19] Ziedonis D Hitsman B Beckham JC Zvolensky M Adler LE Audrain-McGovern J. Tobacco use and cessation in psychiatric disorders: National Institute of Mental Health report. Nicotine Tob Res. (2008) 10:1691–715. 10.1080/14622200802443569 19023823

[20] Devine E Khan RJ Bedford K Jiang WZ Lim H A group based smoking cessation pilot programme for community mental health clients in Sydney. J Smoking Cessation (2014) 9:26–30. 10.1017/jsc.2013.16

[21] Morris CD Waxmonsky JA May MG Tinkelman DG Dickinson M Giese AA. Smoking reduction for persons with mental illnesses: 6-month results from community-based interventions. Community Mental Health J. (2011) 47:694–702. 10.1007/s10597-011-9411-z 21556784

[22] Ziedonis DM George TP. Schizophrenia and nicotine use: report of a pilot smoking cessation program and review of neurobiological and clinical issues. Schizophrenia Bull. (1997) 23:247–54. 9165635 10.1093/schbul/23.2.247

[23] Masuhara JE Heah T Okoli CT Outcomes of a tobacco treatment programme for individuals with severe and persistent mental illness attending a community mental health team. J Smoking Cessation (2014) 9:60–7. 10.1017/jsc.2013.17

[24] Ashton M Rigby A Galletly C. Evaluation of a community-based smoking cessation programme for people with severe mental illness. Tob Control (2013) 24:3. 10.1136/tobaccocontrol-2013-051179 24335478

[25] Okoli CT Mason DA Brumley-Shelton A Robertson H. Providing tobacco treatment in a community mental health setting: a pilot study. J Addict Nurs. (2017) 28:34–41. 10.1097/JAN.0000000000000157 28252509

[26] Dixon LB Medoff D Goldberg R Lucksted A Kreyenbuhl J DiClemente C Is implementation of the 5 A's of smoking cessation at community mental health centers effective for reduction of smoking by patients with serious mental illness? Am J Addict. (2009) 18:386–92. 10.3109/10550490903077747 19874158 PMC5647463

[27] Baker HM Ranney LM Goldstein AO. Pilot implementation of a wellness and tobacco cessation curriculum in north carolina group homes. Commun Mental Health J. (2016) 52:433–8. 10.1007/s10597-015-9975-0 26711097

[28] Lee JG Ranney LM Goldstein AO McCullough A Fulton-Smith SM Collins NO. Successful implementation of a wellness and tobacco cessation curriculum in psychosocial rehabilitation clubhouses. BMC Public Health (2011) 11:702. 10.1186/1471-2458-11-702 21917179 PMC3184072

[29] Rae J Pettey D Aubry T Stol J. Factors affecting smoking cessation efforts of people with severe mental illness: a qualitative study. J Dual Diag. (2015) 11:42–9. 10.1080/15504263.2014.992096 25491704

[30] Knowles S Planner C Bradshaw T Peckham E Man M-S Gilbody S. Making the journey with me: a qualitative study of experiences of a bespoke mental health smoking cessation intervention for service users with serious mental illness. BMC Psychiatry (2016) 16:193. 10.1186/s12888-016-0901-y 27278101 PMC4898392

[31] Burns A LJ Clancy L Strawbridge J Doyle F. Prospective study of provided smoking cessation care in an inpatient psychiatric setting. J Psychiatric Res. (2018) 115:24–31. 10.1016/j.jpsychores.2018.10.006 30470313

[32] Prochaska JJ Hall SE Delucchi K Hall SM. Efficacy of initiating tobacco dependence treatment in inpatient psychiatry: a randomized controlled trial. Am J Public Health (2014) 104:1557–65. 10.2105/AJPH.2013.301403 23948001 PMC4103208

[33] Moore GF Audrey S Barker M Bond L Bonell C Hardeman W. Process evaluation of complex interventions: medical research council guidance. BMJ (2015) 350:1258. 10.1136/bmj.h1258 25791983 PMC4366184

[34] Ritchie J Lewis J Nicholls CM Ormston R editors. Qualitative Research Practice: A Guide for Social Science Students and Researchers. Thousand Oaks, CA: Sage (2013).

[35] Ritchie J Lewis J Elam RG Selecting Samples. Qualitative Research Practice: A Guide for Social Science Students and Researchers. Thousand Oaks, CA: Sage (2013). p. 111.

[36] Braun V Clarke V Using thematic analysis in psychology. Qualit Res Psychol. (2006) 3:77–101. 10.1191/1478088706qp063oa

[37] Trainor K Leavey G. Barriers and Facilitators to Smoking Cessation Among People With Severe Mental Illness: a critical appraisal of qualitative studies. Nicotine Tob Res. (2017) 19:14–23. 10.1093/ntr/ntw183 27613905

[38] Leonardi-Bee J Jayes L O'Mara-Eves A Stansfield C Gibson K Ratschen E Review 5: Barriers to and Facilitators for Smoking Cessation Interventions in Mental Health Services. NICE Evidence Reviews: Smoking Cessation Acute, Maternity and Mental Health Services Draft (2012) 3.

[39] Berg CJ Parelkar PP Lessard L Escoffery C Kegler MC Sterling KL. Defining “smoker”: College student attitudes and related smoking characteristics. Nicotine Tob Res. (2010) 12:963–9. 10.1093/ntr/ntq123 20675365 PMC6407804

[40] Villanti AC Bover Manderski MT Gundersen DA Steinberg MB Delnevo CD. Reasons to quit and barriers to quitting smoking in US young adults. Family Pract. (2016) 33:133–9. 10.1093/fampra/cmv103 26733658 PMC5006105

[41] McCaul KD Hockemeyer JR Johnson RJ Zetocha K Quinlan K Glasgow RE. Motivation to quit using cigarettes: a review. Addict Behav. (2006) 31:42–56. 10.1016/j.addbeh.2005.04.004 15916861

[42] Demonceau J Ruppar T Kristanto P Hughes DA Fargher E Kardas P. Identification and assessment of adherence-enhancing interventions in studies assessing medication adherence through electronically compiled drug dosing histories: a systematic literature review and meta-analysis. Drugs (2013) 73:545–62. 10.1007/s40265-013-0041-3 23588595 PMC3647098

[43] Health Service Executive Policy on E-cigarettes. Available online at: http://www.hse.ie/eng/about/Who/TobaccoControl/campus/ecigpolicy.html (Accessed January 13, 2018].

[44] Parker C McNeill A Ratschen E. Tailored tobacco dependence support for mental health patients: a model for inpatient and community services. Addiction (2012) 107 (Suppl. 2):18–25. 10.1111/j.1360-0443.2012.04082.x 23121356

[45] Lawn S Campion J. Achieving smoke-free mental health services: lessons from the past decade of implementation research. Int J Environ Res Public Health (2013) 10:4224. 10.3390/ijerph10094224 24025397 PMC3799524

[46] Keogan S Burns A Babineau K Clancy L Irish Healthcare staff- Smoking, training and activity in treatment of tobacco dependence -an online survey. Tob Prev Cessation. (2016) 2:73 10.18332/tpc/64946

[47] Keogan S Burns A Babineau K Clancy L Dental practitioners and smoking cessation in Ireland. Tob Prev Cessation (2015) 1:5 10.18332/tpc/59482

[48] Sharpe T Alsahlanee A Ward K Doyle F Systematic Review of Clinician-Reported Barriers to Provision of Smoking Cessation Interventions in Hospital Inpatient Settings. J Smoking Cessation (2018) 13:233–43. 10.1017/jsc.2017.25

[49] Khanna P Clifton AV Banks D Tosh GE. Smoking cessation advice for people with serious mental illness. Cochrane Database Syst Rev. (2016) 1:Cd009704. 10.1002/14651858.CD009704.pub2 26816385 PMC6513396

[50] Peckham E Brabyn S Cook L Tew G Gilbody S. Smoking cessation in severe mental ill health: what works? an updated systematic review and meta-analysis. BMC Psychiatry (2017) 17:252. 10.1186/s12888-017-1419-7 28705244 PMC5513129

[51] Stead LF Buitrago D Preciado N Sanchez G Hartmann-Boyce J Lancaster T Physician advice for smoking cessation. Cochrane Database Syst Rev. (2013) CD000165 10.1002/14651858.CD000165.pub4 23728631 PMC7064045

[52] Harker K Cheeseman H Shifting culture and taking action to reduce smoking and premature death among people with a mental health condition. J Public Mental Health (2016) 15:184–7. 10.1108/JPMH-09-2016-0046

[53] Rice VH Hartmann-Boyce J Stead LF. Nursing interventions for smoking cessation. Cochrane Database Syst Rev. (2013) 12:Cd001188. 10.1002/14651858.CD001188.pub4 23939719

[54] Solway ES. The lived experiences of tobacco use, dependence, and cessation: insights and perspectives of people with mental illness. Health Soc Work (2011) 36:19–32. 10.1093/hsw/36.1.19 21446606

[55] Snyder M McDevitt J Painter S. Smoking cessation and serious mental illness. Arch Psychiatr Nurs. (2008) 22:297–304. 10.1016/j.apnu.2007.08.007 18809122

[56] Kerr S Woods C Knussen C Watson H Hunter R. Breaking the habit: a qualitative exploration of barriers and facilitators to smoking cessation in people with enduring mental health problems. BMC Public Health (2013) 13:221. 10.1186/1471-2458-13-221 23497231 PMC3599988

[57] Twyman L Bonevski B Paul C Bryant J. Perceived barriers to smoking cessation in selected vulnerable groups: a systematic review of the qualitative and quantitative literature. BMJ Open (2014) 4:e006414. 10.1136/bmjopen-2014-006414 25534212 PMC4275698

[58] McNeill A editor Smoking and Mental Health: A Review of the Literature. SYMPOSIUM Report: Smoking and Mental Health Smoke-free. London; Citeseer (2001).

[59] Lucksted A Dixon LB Sembly JB. A focus group pilot study of tobacco smoking among psychosocial rehabilitation clients. Psychiatr Serv. (2000) 51:1544–8. 10.1176/appi.ps.51.12.1544 11097651

[60] Missen RL Brannelly T Newton-Howes G. Qualitative exploration of family perspectives of smoke-free mental health and addiction services. Int J Ment Health Nurs. (2013) 22:294–303. 10.1111/j.1447-0349.2012.00882.x 23066762

[61] Esterberg ML Compton MT. Smoking behavior in persons with a schizophrenia-spectrum disorder: a qualitative investigation of the transtheoretical model. Soc Sci Med. (2005) 61:293–303. 10.1016/j.socscimed.2004.11.057 15893046

[62] Banham L Gilbody S. Smoking cessation in severe mental illness: what works? Addiction (2010) 105:1176–89. 10.1111/j.1360-0443.2010.02946.x 20491721

[63] Scott D Happell B. The high prevalence of poor physical health and unhealthy lifestyle behaviours in individuals with severe mental illness. Issues Mental Health Nurs. (2011) 32:589–97. 10.3109/01612840.2011.569846 21859410

[64] Williams JM Ziedonis D Addressing tobacco among individuals with a mental illness or an addiction. Addict Behav. (2004) 29:1067–83. 10.1016/j.addbeh.2004.03.009 15236808

[65] Peckham E Arundel C Bailey D Brownings S Fairhurst C Heron P. Smoking Cessation Intervention for Severe Mental Ill Health Trial (SCIMITAR+): study protocol for a randomised controlled trial. Trials (2017) 18:44. 10.1186/s13063-017-1789-7 28126031 PMC5270301

[66] Vilardaga R Rizo J Kientz JA McDonell MG Ries RK Sobel K. User experience evaluation of a smoking cessation app in people with serious mental illness. Nicotine Tob Res. (2016) 18:1032–8. 10.1093/ntr/ntv256 26581430 PMC4900234

[67] Roberts SH Bailey JE. An ethnographic study of the incentives and barriers to lifestyle interventions for people with severe mental illness. J Adv Nurs. (2013) 69:2514–24. 10.1111/jan.12136 23621276

[68] Lewis J Ritchie J Generalising from Qualitative Research. Qualitative Research Practice: A Guide for Social Science Students and Researchers. Thousand Oaks, CA: Sage (2003). p. 347–62.

[69] Patton M Qualitative Research and Evaluation Methods. Thousand Oaks, CA: Sage (2002).

[70] Lambert SD Loiselle CG. Combining individual interviews and focus groups to enhance data richness. J Adv Nurs. (2008) 62:228–37. 10.1111/j.1365-2648.2007.04559 18394035

[71] Ritchie J Lewis J. Qualitative Research Practice. London: Sage (2003).

